# Supplementary material for: NF-κB inhibition rescues cardiac function by remodeling calcium genes in a Duchenne muscular dystrophy model
Source: Nat Commun. 2018 Aug 24;9:3431. doi: 10.1038/s41467-018-05910-1 (PMC6109146; doi:10.1038/s41467-018-05910-1)
Supplement: Supplementary file 1 — Supplementary Information [file 41467_2018_5910_MOESM1_ESM.pdf]

NF-κB inhibition rescues cardiac function through chromatin remodeling of calcium genes in a model of Duchenne muscular dystrophy

Peterson et al.

Supplementary information

Supplementary Figures

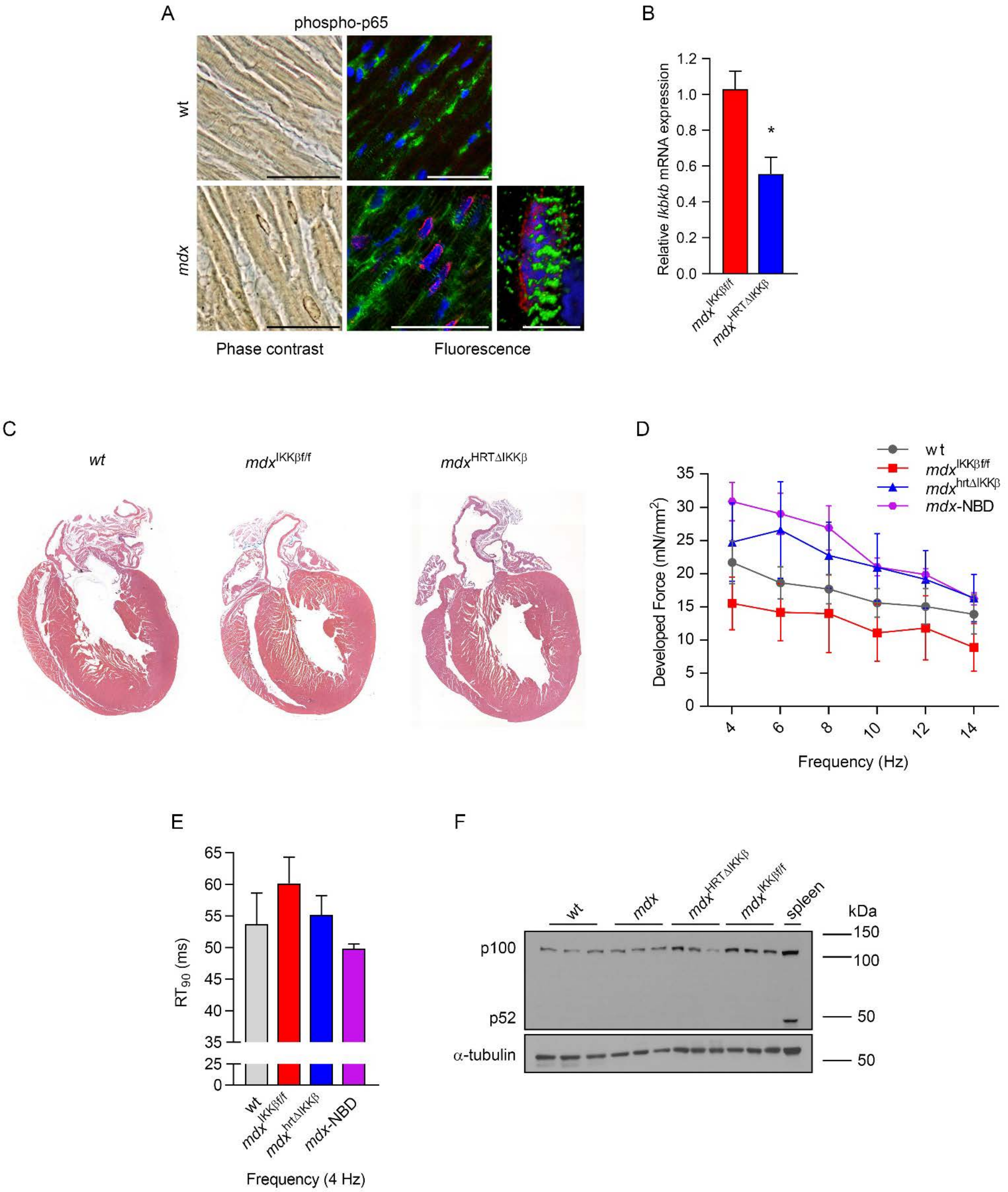

Supplementary Fig 1. Supporting cardiac images, PCR, physiology, and western blot
(**A**) Representative images of phosho-p65 staining from hearts heart sections. Left panels were captured with phase contrast microscopy to enhance visualization of myofibers. Phospho-p65 positive nuclei are labeled brown. Middle and right panels, immunofluorescence labeling of phospho-p65 positive nuclei (red) and alpha-sarcomeric actinin (green) sections. Scale bar = 50μm for left and middle panels and 10μm for right panel. (**B**) qPCR to confirm reduced *Ikbkb* gene expression in genetic knockout mice. Total RNA isolated from whole hearts (n = 6 $mdx^{IKK\beta f/f}$; 4 $mdx^{HRT\Delta IKK\beta}$). (**C**) Representative 1-year-old whole heart H&E sections. (**D**) Basal developed force measured from isolated multicellular cardiac muscles of 7-month old mice (n = 7 wt; 6 $mdx^{IKK\beta f/f}$; 9 $mdx^{HRT\Delta IKK\beta}$; 3 NBD treated (*mdx*-NBD). (**E**) Basal relaxation time (RT_{90}) measured from multicellular cardiac muscles (n = 7 wt; 7 $mdx^{IKK\beta f/f}$; 8 $mdx^{HRT\Delta IKK\beta}$; 3 *mdx*-NBD). (**F**) Western blot performed on whole tissue lysates and probed for p100/p52 and α-tubulin (used as a loading control). Data expressed as means ± SEM. (**B**) * $p < 0.05$ by 2-tailed Student's t test (**D**) $p = 0.359$ (main effects for genotype/treatment), by 2-way repeated measures ANOVA. (**E**) $p = 0.488$, by 1-way ANOVA.

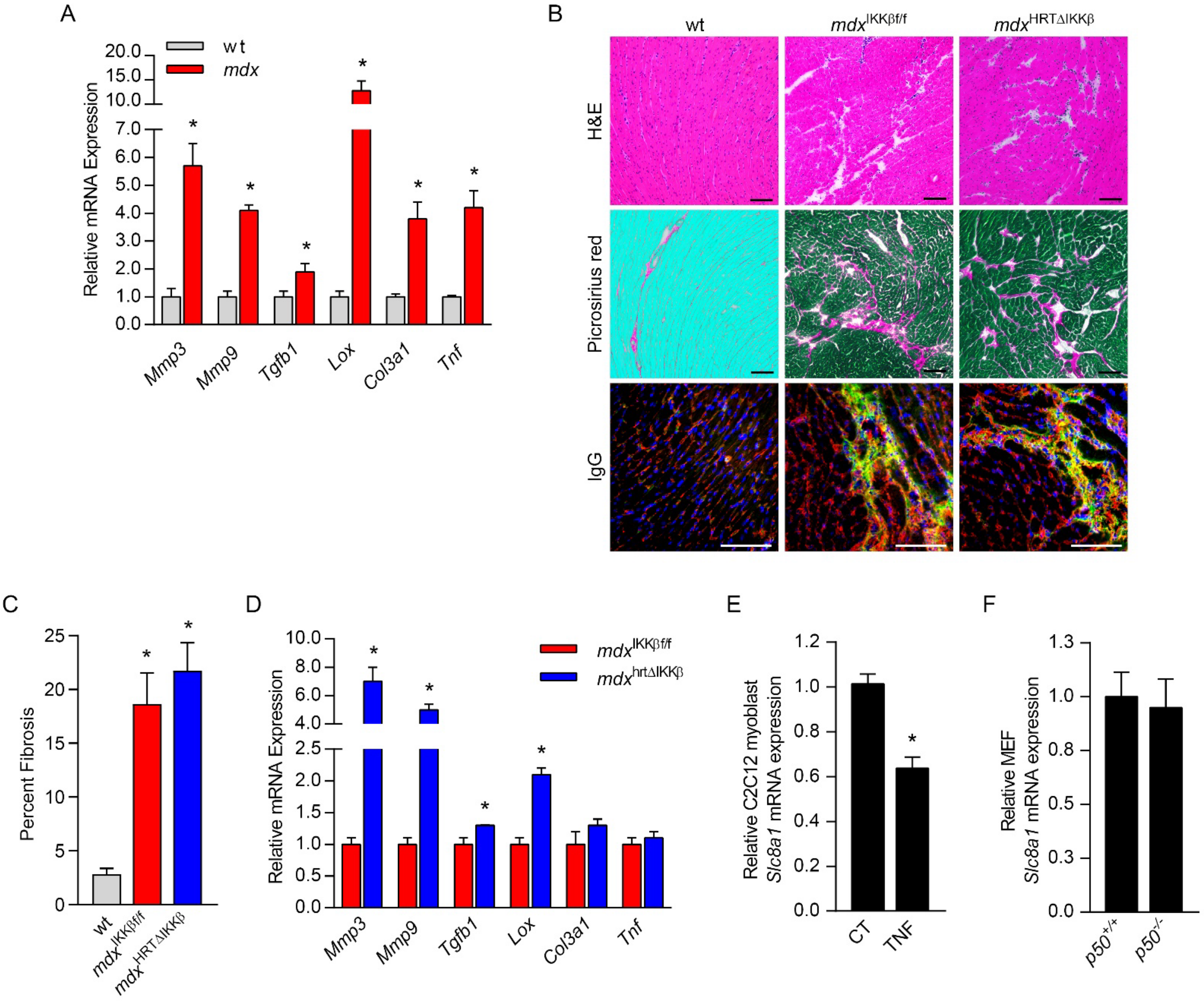

Supplementary Fig 2. Cardiac fibrosis and *Slc8a1* expression in a subset of cells
(**A**) Gene expression analyzed by qPCR on total RNA isolated from 3-month-old whole hearts (n = 5 except n = 4 *Mmp3*, *Mmp9*). (**B**) Representative images of H&E, Picrosirius Red, and Laminin (red) with IgG (green) staining prepared from 1-year-old heart sections. (**C**) Quantitation of fibrosis in sections from 1-year-old hearts (n = 5). (**D-F**) Gene expression analyzed by qPCR on total RNA isolated from (**D**) 6-month-old whole hearts (n = 6 $mdx^{IKK\beta f/f}$; n =10 $mdx^{HRT\Delta IKK\beta}$), (**E**) C2C12 myoblasts untreated (CT) and treated with TNF (n = 8) and (**F**) Mouse embryonic fibroblasts (MEFs) wild-type ($p50^{+/+}$) and null ($p50^{-/-}$) for the p50 subunit of NF-κB (n = 5 $p50^{+/+}$; 4 $p50^{-/-}$). Scale bar = 100μm. Data expressed as means ± SEM. (**A, D, E**) * $p < 0.05$ wt, $mdx^{IKK\beta f/f}$, or CT by 2-tailed Student's t test. (**C**) * $p < 0.05$ wt by 1-way ANOVA followed by Tukey multiple comparison test. (**F**) p = 0.78, by 2-tailed Student's t test.

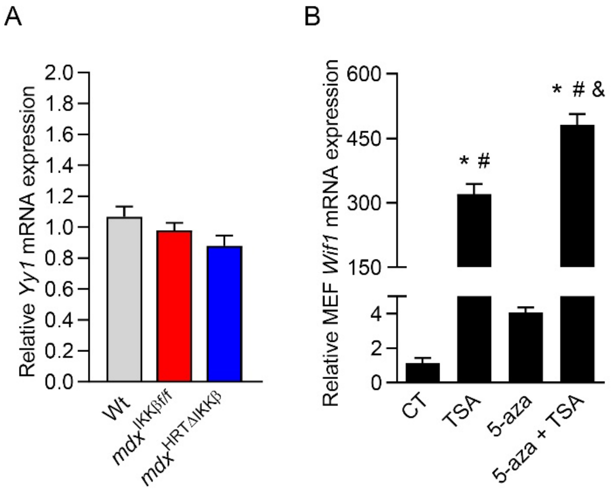

Supplementary Fig 3. *Yy1* expression and chromatin-mediated changes with *Wif1*
(**A-B**) qPCR analysis performed on total RNA isolated from (**A**) 6-7-month-old hearts (n = 6 wt; 5 $mdx^{IKK\beta f/f}$ and $mdx^{HRT\Delta IKK\beta}$) and (**B**) MEFs that were vehicle treated (CT), treated with trichostatin A (TSA), 5-Aza-2′-Deoxycytidine (5-aza) treated, and treated with a combination of 5-aza and TSA (n = 5 CT; 6 TSA; 3 5-aza; 4 5-aza + TSA). Data expressed as means ± SEM. (**A**) $p = 0.140$, by 1-way ANOVA and (**B**) * $p < 0.05$ CT; # $p < 0.05$ 5-aza; & $p < 0.05$ TSA, by 1-way ANOVA followed by Tukey Post-hoc analysis.

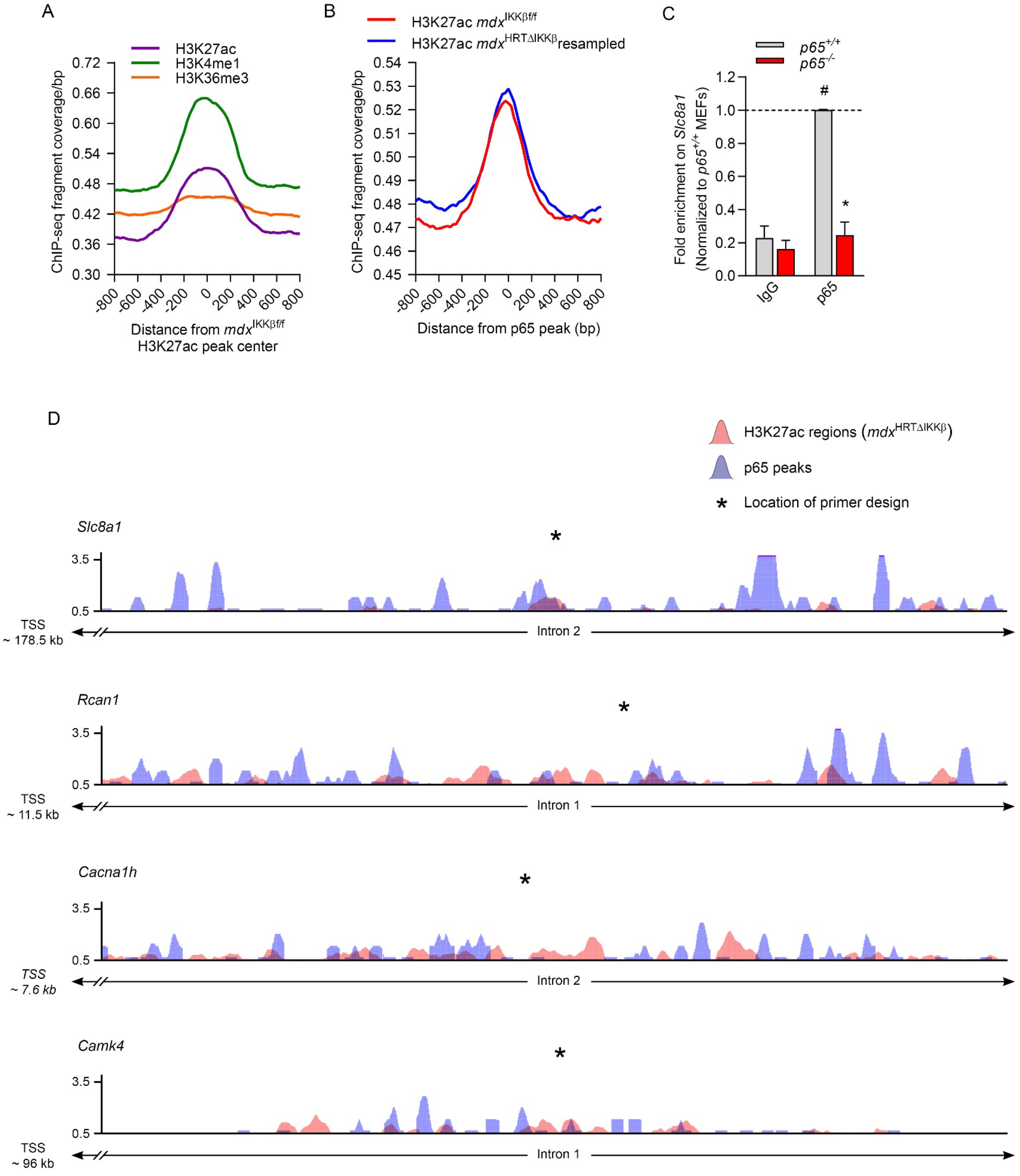
A
H3K27ac
H3K4me1
H3K36me3
ChIP-seq fragment coverage/bp
0.72
0.66
0.60
0.54
0.48
0.42
0.36
0.30
-800 -600 -400 -200 0 200 400 600 800
Distance from mdxIKKβf/f H3K27ac peak center
B
H3K27ac mdxIKKβf/f
H3K27ac mdxHRTΔIKKβresampled
ChIP-seq fragment coverage/bp
0.54
0.53
0.52
0.51
0.50
0.49
0.48
0.47
0.46
0.45
-800 -600 -400 -200 0 200 400 600 800
Distance from p65 peak (bp)
C
p65+/+
p65-/-
Fold enrichment on Slc8a1 (Normalized to p65+/+ MEFs)
1.2
1.0
0.8
0.6
0.4
0.2
0.0
#
*
IgG
p65
D
H3K27ac regions (mdxHRTΔIKKβ)
p65 peaks
* Location of primer design
Slc8a1
3.5
0.5
TSS ~ 178.5 kb
Intron 2
Rcan1
3.5
0.5
TSS ~ 11.5 kb
Intron 1
Cacna1h
3.5
0.5
TSS ~ 7.6 kb
Intron 2
Camk4
3.5
0.5
TSS ~ 96 kb
Intron 1

Supplementary Fig 4. Bioinformatics analysis on hearts and p65 ChIP in MEFs
(**A**) Genome-wide fragment density, showing potential overlap of (**A**) ChIP-seq histone marks across peaks from our *mdx*$^{IKK\beta f/f}$ H3K27ac ChIP-seq performed in hearts (**B**) *mdx*$^{IKK\beta f/f}$ and *mdx*$^{HRT\Delta IKK\beta}$ ChIP-seqs across peaks from p65 ChIP-seq that were normalized to control for the differing number of reads in each ChIP-seq. (**C**) qPCR analysis to detect enrichment in the *Slc8a1* regulatory region. DNA from ChIPs performed with a p65 antibody on MEFs that were wild-type (*p65*$^{+/+}$) or null (*p65*$^{-/-}$) for p65 (n = 3). (**D**) Images visualizing H3K27ac *mdx*$^{HRT\Delta IKK\beta}$ and p65 ChIP-seqs. Peaks were visualized across denoted genes. Data are expressed as means ± SEM. * $p < 0.05$ *p65*$^{+/+}$ MEF, # $p < 0.05$ IgG, by 2-way ANOVA followed by Tukey Post-hoc analysis.

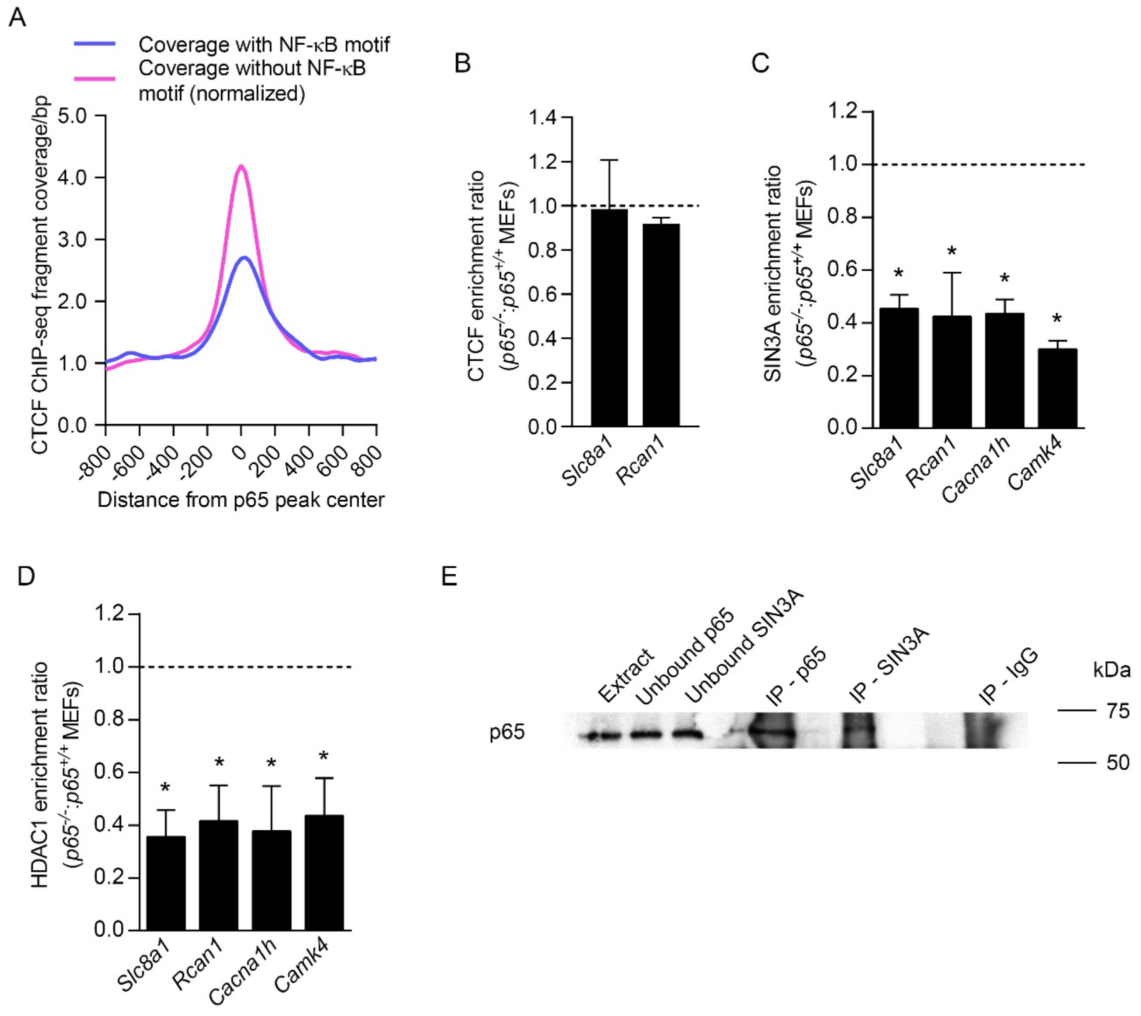

Supplementary Fig 5. Bioinformatics on CTCF, ChIP for repressor proteins, co-immunoprecipitation for SIN3A

(**A**) Genome-wide fragment density, showing potential overlap of CTCF across p65 peaks that were split into groups that either contained or lacked a consensus NF-κB binding site. Data was normalized to control for the differing number of reads in each split peak group. (**B-D**) qPCR analysis to detect enrichment in denoted genes. DNA from ChIPs was performed on MEFs that were wild-type (*p65*$^{+/+}$) or null (*p65*$^{-/-}$) for p65 with a (**B**) CTCF antibody (n = 3), (**C**) SIN3A antibody (n = 3) and (**D**) HDAC1 antibody (n = 3). Genes expressed as a ratio. Dotted line represents level of enrichment equal to *p65*$^{+/+}$ MEFs. Bars represent depletion in *p65*$^{-/-}$ MEFs. (**E**) Immunoblot for p65 following co-immunoprecipitation. First three lanes are controls (whole extract, unbound p65, unbound SIN3A). Other labeled lanes were immunoprecipitated (IP) with p65, SIN3A, or IgG (as an additional control). Unlabeled lanes were not loaded with sample. Data expressed as means ± SEM. (**B**) p = 0.947 *Slc8a1*; p = 0.094 *Rcan1* and (**C-D**) * $p < 0.05$ *p65*$^{+/+}$ by 2-tailed Student's t test.

Blots from figure 1

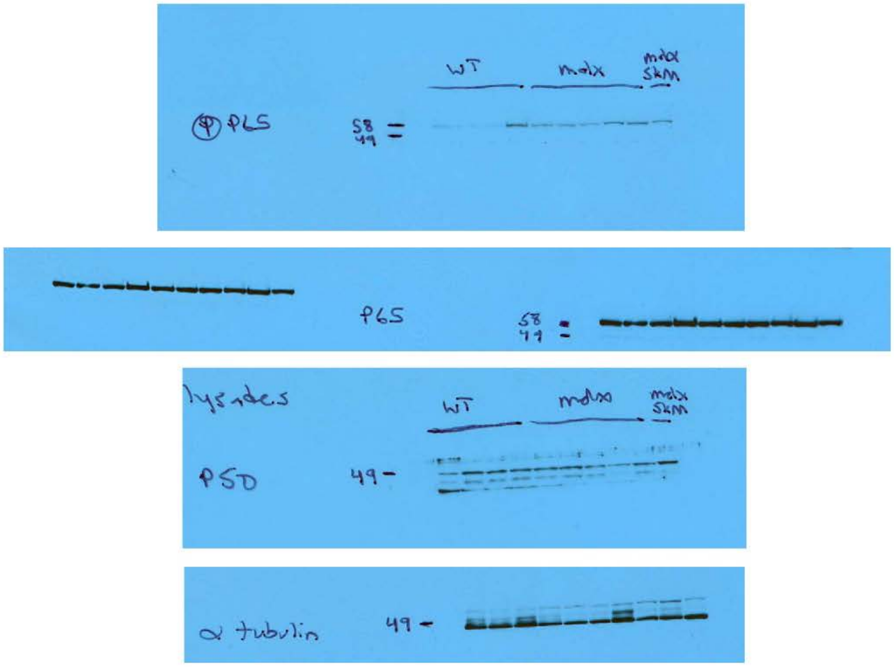

Blots from supplementary figure 1

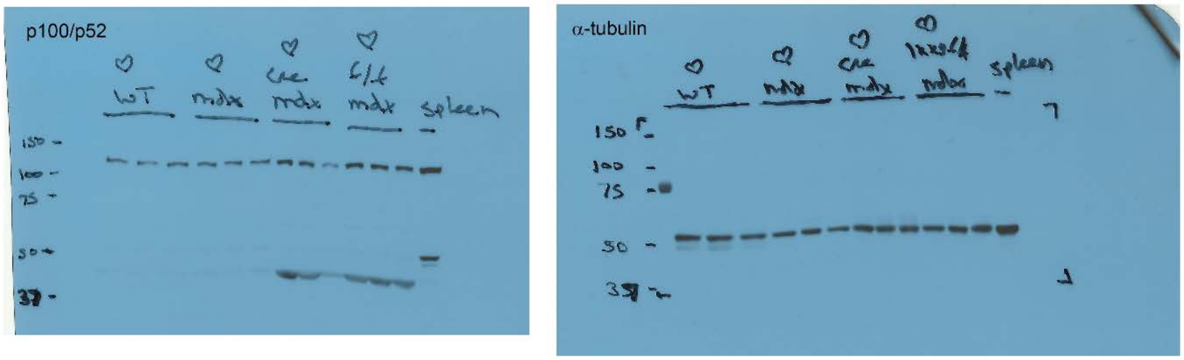

IP from supplementary figure 5

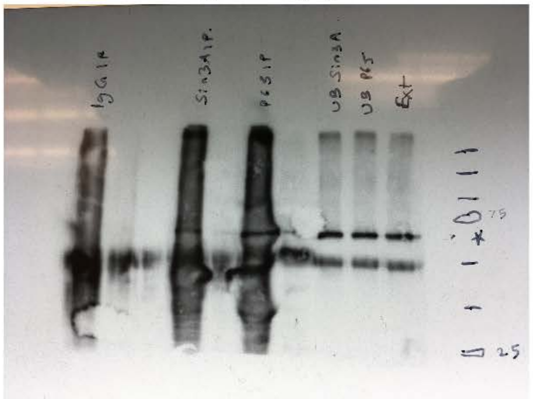

Supplementary Fig 6. Uncropped blots

Supplementary Tables

Pressure volume loop parameters	wt	$mdx^{IKK\beta f/f}$	$mdx^{HRT\Delta IKK\beta}$	P value
ESPVR	6.7 ± 1.0 (n = 10)	7.0 ± 0.9 (n = 21)	8.3 ± 1.2 (n = 12)	0.548
PRSW	53.0 ± 8.6 (n = 10)	67.4 ± 5.5 (n = 22)	74.4 ± 5.3 (n = 13)	0.1184
dp/dt_{max} (mm Hg ms-1)				
360 BPM	11057 ± 492.1 (n = 3)	10247 ± 705.3 (n = 8)	9966 ± 588.9 (n = 7)	0.6592
600 BPM	10061 ± 830.3 (n = 3)	8279 ± 675.5 (n = 6)	7027 ± 876.3 (n = 5)	0.0994
Dobutamine	15737 ± 1990.0 (n = 3)	11480 ± 959.8 (n = 8)	12459 ± 556.9 (n = 7)	0.062
dp/dt_{min} (mm Hg ms-1)				
360 BPM	-6683 ± 477.7 (n = 3)	-6408 ± 604.6 (n = 8)	-6178 ± 587.4 (n = 5)	0.8968
600 BPM	-6138 ± 815.2 (n = 3)	-5661 ± 797.1 (n = 6)	-4899 ± 505.3 (n = 5)	0.555
Dobutamine	-7646 ± 1748 (n = 3)	-5853 ± 809.2 (n = 8)	-6350 ± 577.8 (n = 5)	0.4962
Systolic pressure (mm Hg)				
360 BPM	144 ± 11.6 (n = 6)	137 ± 8.7 (n = 8)	130 ± 8.3 (n = 8)	0.6128
600 BPM	126 ± 11.4 (n = 5)	106 ± 9.4 (n = 8)	91 ± 7.9 (n = 8)	0.0682
Dobutamine	141 ± 7.7 (n = 6)	112 ± 8.3 (n = 8)	127 ± 8.0 (n = 8)	0.0694
Diastolic pressure (mm Hg)				
360 BPM	19 ± 4.5 (n = 6)	15 ± 2.8 (n = 8)	19 ± 2.2 (n = 8)	0.4682
600 BPM	19 ± 3.8 (n = 5)	17 ± 2.8 (n = 8)	21 ± 2.8 (n = 8)	0.5705
Dobutamine	17 ± 4.6 (n = 6)	20 ± 2.4 (n = 8)	18 ± 2.6 (n = 8)	0.7695
Contractility index ($dP/dt_{max}/EDV$)				
360 BPM	233 ± 54.8 (n = 3)	228 ± 22.6 (n = 8)	170 ± 22.1 (n = 7)	0.213
600 BPM	233 ± 40.9 (n = 3)	203 ± 23.1 (n = 6)	143 ± 15.1 (n = 5)	0.0846
Dobutamine	377 ± 108.1 (n = 2)	252 ± 14.3 (n = 8)	249 ± 32.3 (n = 7)	0.1168

Supplementary Table 1. Data are expressed as means ± SEM

Primer	Anneal temp	Direction	Sequence
Ikbkb	60	Forward	ATCAAGCAATGCCGACAGGA
		Reverse	ATGGCCAGCAGTGGCAAAT
Slc8a1	60	Forward	TGCTCTTGGAACCTCGGTGC
		Reverse	AGAAAGCTAGCGTGCCGGGG
Rcan1	60	Forward	GCCCAATCCCGACAAACAGT
		Reverse	CTCACACACGTGGACCACCA
Cacna1h	60	Forward	CCGCTCTCTGAGCCTCTCAC
		Reverse	AAAGTGTGCTTGCGGGTACG
Camk4	60	Forward	AAAGCGGCCAACTTTGTTCA
		Reverse	GTCCTTGGCATCTTGGGTTG
mmp3	58	Forward	GGAAATCCCACATCACCTACAGG
		Reverse	CCTCATATGCAGCATCCATGT
mmp9	55	Forward	CTGGTGATCTCTTCTAGAGACTGG
		Reverse	CACAGTCTGACCTGAACCATAA
Tgfb1	53	Forward	TGCTGCCTTCGCCCTCTTTAC
		Reverse	AAGCGGAAGCTTCGGGATTT
Lox	60	Forward	AACAACGGGCAGGTGTTCAGT
		Reverse	GGTTGTCACGCAGCAGAAGAA
Col3a1	60	Forward	AACCAGGTGCTAAAGGAGAAAGAGG
		Reverse	TGGATTACCATTATTGCCAGGAGGA
Tnf	60	Forward	CCCAAAGGGATGAGAAGTTCCC
		Reverse	CCTGGTATGAGATAGCAAATCGG
Wif1	60	Forward	CGGCAAACAAGACGGGGTAG
		Reverse	GAGGCCCGTAGAACCCATCC
Gapdh	60	Forward	AGCCTCGTCCCGTAGACAAAA
		Reverse	GCCTTGACTGTGCCGTTGAAT

Supplementary Table 2. SYBR Green cDNA primer sequences and conditions

Primer	Anneal temp	Direction	Sequence	Roche probe
Slc8a1	56	Forward	AAGAGATCAGTTCCCGTCTCC	26
Original - cells		Reverse	AAGGGGATCCATCCACAAG	
Slc8a1	56	Forward	CACCTCCAACAGGACAAAGTT	106
Original - Hearts		Reverse	CTTAGCCTGGCTCCCTGAA	
Slc8a1	56	Forward	ACACGATGTCTGTAATGACAATGA	60
From ChIP-seq		Reverse	TGCTGGAGGCTTTGTCAAC	
Rcan1	56	Forward	AAAGGAGGGGATGTGTTGG	13
		Reverse	TTCAACTCAGGGCCAGAAAG	
Cacna1h	56	Forward	ACCTGAAATGGTTACCCAAGC	20
		Reverse	AGCCAGGGCTTGTTATAATGC	
Camk4	56	Forward	CCACAACAGTTCGTAGAAAACG	5
		Reverse	TGGGCCCACACAAACTGTA	

Supplementary Table 3. Taqman DNA primer sequences and conditions

Supplementary Methods

Tissue preparation and histology Hearts were either snap frozen for RNA and protein preparations, mounted in OCT and frozen in 2-Methylbutane cooled to the temperature of liquid nitrogen, or formalin-fixed, paraffin-embedded (FFPE; for whole heart sections). For frozen tissue, 10 μm sections were prepared on a cryostat. For FFPE, 8 μm sections were prepared on a microtome. Histological staining was performed for H&E and picrosirius red (PSR). PSR slides were counter-stained with Fast Green (FG). Fibrosis was quantitated using ImageJ software with the color deconvolution plugin. Fibrosis was determined as a percentage of PSR positive area relative to total area [1]. Primary antibodies: laminin (1:500; L9393 Sigma), phosphorylated p65 serine 536 (1:500; 3031 Cell Signaling), alpha-sarcomeric actinin (1:500; A2172 Sigma). Secondary antibodies (1:250): for immunofluorence Alexa Fluor IgG (594; A11037 and 488; A11029) and IgM (488; A21042) (Molecular probes), for immunohistochemistry HRP goat anti-rabbit IgG (1:250; PI1000 Vector) secondary antibody and a DAB substrate kit (SK4100 Vector) was used.

Western blotting Protein was extracted and standard immunoblotting procedures were performed. Primary antibody: p100/p52 (1:1000; 4882 Cell Signaling).

ChIP ChIP was performed as described in the main manuscript. Antibody: 3 μg p65 (17-10060).

ChIP-sequencing analysis The UCSC Genome Browser (http://genome.ucsc.edu/) was used to visualize ChIP-seq peaks [2]. MultiWigs were created as outlined on the UCSC website using the mm9 assembly.

Immunoprecipitation Whole-cell extracts were prepared from MEFs in RIPA buffer. Approximately 300 μgs of extract was used for each immunoprecipitation with 2 μgs of antibody. Antibodies: p65 (372) and SIN3A (7691) (Cell Signaling). Antibody against IgG was used for the negative control. After an

overnight incubation, antibody-antigen complexes were collected with protein A agarose beads (Invitrogen). Beads were washed with RIPA buffer and resuspended in protein loading dye. Immunoprecipitated samples with appropriate controls were resolved by SDS–polyacrylamide gel electrophoresis and transferred onto PVDF membranes for Western blotting analysis. Antibody: p65 (1:1000; 109 Santa Cruz).

Supplementary References

1 Schneider, C. A., Rasband, W. S. & Eliceiri, K. W. NIH Image to ImageJ: 25 years of image analysis. *Nature methods* **9**, 671-675 (2012).

2 Raney, B. J. *et al.* Track data hubs enable visualization of user-defined genome-wide annotations on the UCSC Genome Browser. *Bioinformatics* **30**, 1003-1005, (2014).
